# Replisome Assembly at Bacterial Chromosomes and Iteron Plasmids

**DOI:** 10.3389/fmolb.2016.00039

**Published:** 2016-08-11

**Authors:** Katarzyna E. Wegrzyn, Marta Gross, Urszula Uciechowska, Igor Konieczny

**Affiliations:** Department of Molecular and Cellular Biology, Intercollegiate Faculty of Biotechnology of University of Gdansk and Medical University of GdanskGdansk, Poland

**Keywords:** replication initiation, DnaA, Rep, iteron plasmids, replisome assembly

## Abstract

The proper initiation and occurrence of DNA synthesis depends on the formation and rearrangements of nucleoprotein complexes within the *origin* of DNA replication. In this review article, we present the current knowledge on the molecular mechanism of replication complex assembly at the *origin* of bacterial chromosome and plasmid replicon containing direct repeats (iterons) within the *origin* sequence. We describe recent findings on chromosomal and plasmid replication initiators, DnaA and Rep proteins, respectively, and their sequence-specific interactions with double- and single-stranded DNA. Also, we discuss the current understanding of the activities of DnaA and Rep proteins required for replisome assembly that is fundamental to the duplication and stability of genetic information in bacterial cells.

## Introduction

The replication of genetic material is one of the most fundamental processes that influence the proper functioning of each living cell. The synthesis of new DNA molecule, in case of both bacterial chromosomes and plasmids, starts at a well-defined place called *origin* and can be divided into the following steps: (1) *origin* recognition by replication initiation proteins and open complex formation (2) helicase loading, activation and primer synthesis (3) replisome assembly and DNA synthesis. Although these main steps during the DNA replication process are common, when considering replication of bacterial chromosomes and iteron plasmids replicated by theta mechanism, some differences can be observed (Table 1).

**Table 1:** **Comparison of general features of iteron plasmid and chromosomal DNA replication initiation**.

	**Bacterial chromosome**	**Iteron plasmids**
Replication initiator	DnaA	Rep, DnaA
		
Binding sites for initiator	Strong and weak DnaA-boxes	DnaA-boxes for DnaA
		Iterons for Rep
		Weak binding sites ?
		
DNA binding domain of initiator	DNA binding domain (DBD) AAA+ domain	Winged Helix domain (WH)
		
Nucleotide binding by initiator	+	Rep protein ?
		DnaA protein ?
		
Oligomer formation by initiator protein	+	Rep oligomers ?
		Rep-DnaA oligomers ?
		
Binding of initiator to dsDNA	+	Rep protein +
		DnaA protein + (in *Pseudomonas* spp. DnaA is dispensable)
		
Binding of initiator to ssDNA	+	Rep protein +
		DnaA protein ?
		
Assistance of architectural proteins: IHF, HU	More efficient *oriC-*dependent DNA replication	More efficient plasmid *origin-*dependent DNA replication
		HU is required for replication of some plasmids
		
Interaction of initiator with helicase DnaB	+	Rep +
		DnaA +

A DNA replication process of chromosome and plasmid DNA starts when Origin Binding Proteins (OBP) recognize and bind specific motifs located within *origin* region. Despite the differences in structure of bacterial and plasmid initiators, DnaA and Rep proteins, respectively, they have the same function. Binding of initiators results in a modulation of nearby DNA topology and opening of double-stranded helix structure in DNA unwinding element (DUE). A single-stranded DUE region becomes a place where helicase is loaded. In the next step the replisome is assembled and holoenzyme of DNA Polymerase III can play its role during DNA synthesis.

Despite many years of research on DNA replication, new aspects of this process are still being discovered. Recently, the novel activities of replication initiator proteins have been shown. However, especially in case of plasmid DNA replication, there are many questions concerning the replication initiation and replisome assembly that still need to be answered.

## Origin recognition and open complex formation by replication initiation proteins

### Origin recognition and open complex formation by chromosomal initiator at chromosomal *origin*

**Figure 1:**
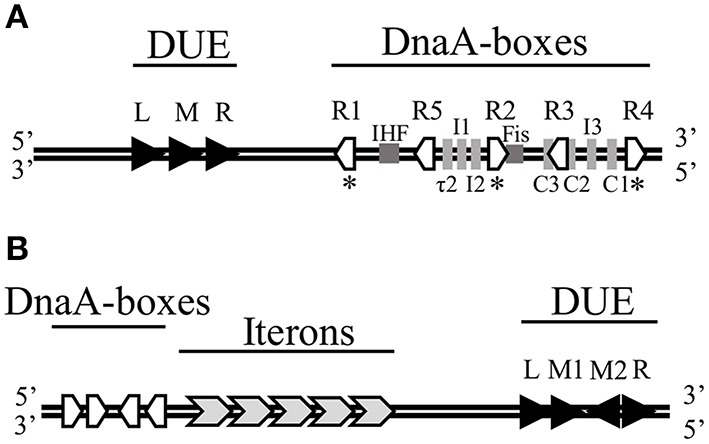
**The minimal ***origins*** of DNA replication of (A) ***E. coli*** chromosome and (B) RK2 plasmid. (A)** The genetic organization of *E. coli oriC* comprises 13-mers within the DNA Unwinding Element (DUE) and DnaA-boxes as well as binding sites of IHF and Fis proteins. Asterisks (^*^) below the *oriC* indicate strong DnaA-boxes. **(B)** The genetic organization of RK2 plasmid *oriV* consisting of DnaA-boxes, Iterons, and DUE. Black arrows mark 13-mers.

**Figure 2:**
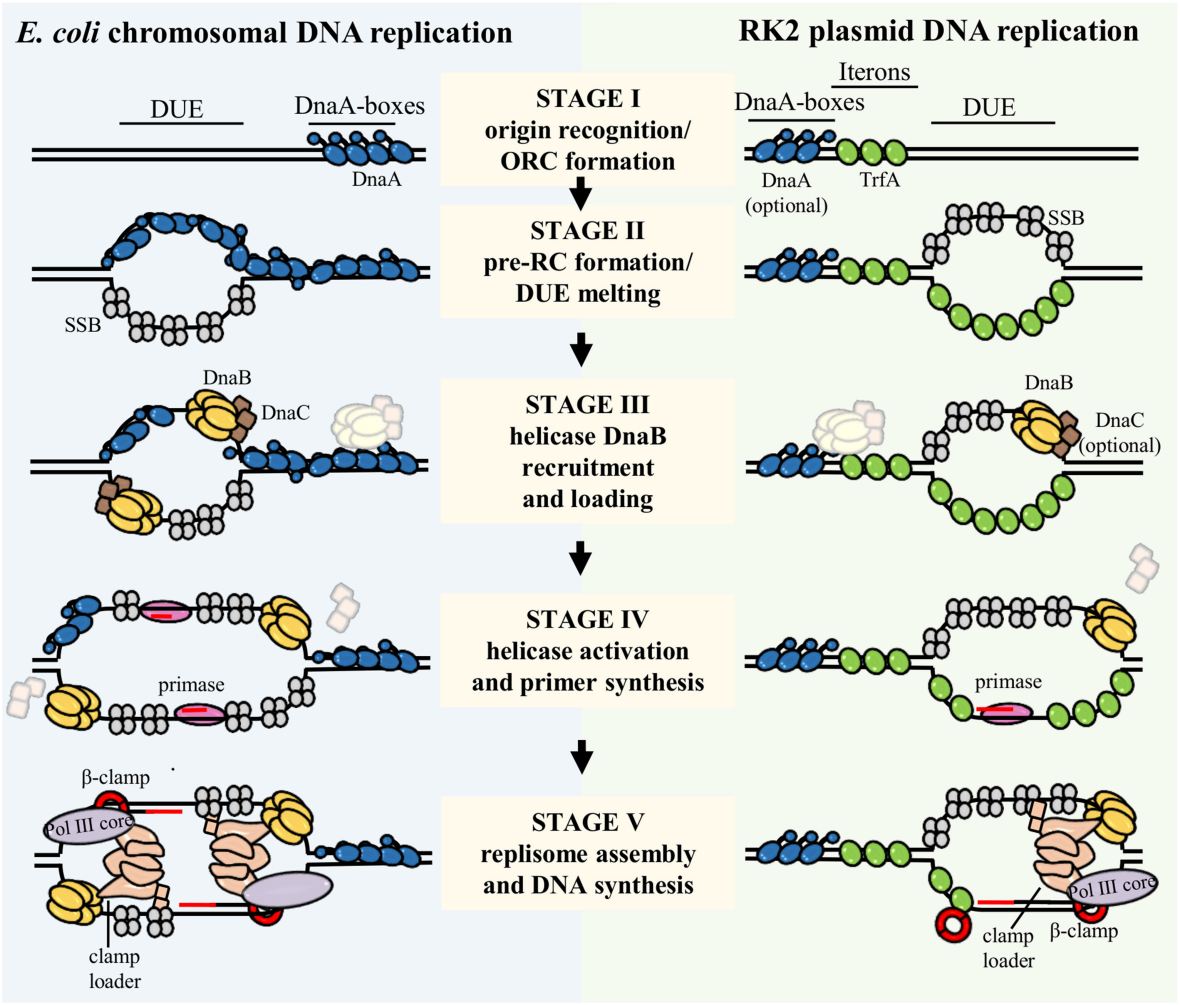
**The process of bacterial chromosome and plasmid DNA replication initiation and replisome assembly**. The scheme presents replication initiation and replisome assembly at chromosomal *E. coli origin, oriC* (left), and RK2 plasmid *origin, oriV* (right). The DNA replication initiation starts with binding a replication initiator(s) DnaA and TrfA to the DnaA boxes and Iterons, respectively (Stage I). Origin Recognition Complex (ORC) formation induces local destabilization and pre-Replication Complex (pre-RC) formation and melting of the DNA Unwinding Element (DUE) region (Stage II). Then, assisted by replication initiators and the DnaC helicase loader, the DnaB helicase is recruited and loaded onto the single-stranded DUE (Stage III). In case of plasmid DNA replication the requirement for DnaA and DnaC is optional as it depends on the host organism. Association of DnaG primase triggers the release of helicase loader, helicase activation and primers synthesis (Stage IV). Next, the holoenzyme of DNA Polymerase III, which comprises clamp loader, DNA Polymerase III core (Pol III core), and β-clamp is assembled and conducts DNA synthesis (Stage V). Lagging strand synthesis was omitted for simplicity. Proteins involved in described stages of DNA replication initiation and replisome assembly processes are depicted in the scheme. IHF and Fis were omitted in this scheme.

The very first step of replication initiation process is the recognition of specific motifs located within the *origin* region of DNA molecule (Figure 1) by replication initiation proteins (Figure 2, Stage I). The bacterial chromosome replication initiator DnaA protein consists of four domains, which play distinct roles (Sutton and Kaguni, 1997, Figure 3A). The best characterized DnaA is the *Escherichia coli* protein (*Ec*DnaA), although structural data is limited only to domain I (resolved by NMR-analysis; Abe et al., 2007b) and IV (resolved in a nucleoprotein complex by crystallography; Fujikawa et al., 2003). Information concerning the structure of DnaA initiator is supplemented by structure of domains I and II of *Mycoplasma genitalium* DnaA (*Mg*DnaA; Lowery et al., 2007), domains I and II of *Helicobacter pylori* DnaA (*Hp*DnaA) in a complex with HobA protein (Natrajan et al., 2009), domains III and IV of *Aquifex aeolicus* DnaA (*Aa*DnaA; Erzberger et al., 2002, 2006), domain III of *Thermatoga maritima* DnaA (*Tm*DnaA; Ozaki et al., 2008), and domain IV of *Mycobacterium tuberculosis* (*Mt*DnaA; Tsodikov and Biswas, 2011). Domain I of *Ec*DnaA, located at the N-terminus of the protein, was shown to be involved in oligomerization of DnaA (Weigel et al., 1999; Simmons et al., 2003; Abe et al., 2007a), helicase loading (Sutton et al., 1998; Seitz et al., 2000), and interaction with DiaA (Keyamura et al., 2007), HU (Chodavarapu et al., 2008a), Dps (Chodavarapu et al., 2008b), and ribosomal protein L2 (Chodavarapu et al., 2011). The interaction with DiaA homologe, HobA protein, was shown for domains I and II of *Hp*DnaA (Natrajan et al., 2007, 2009; Zawilak-Pawlik et al., 2007). In *Bacillus subtilis*, domain I of DnaA (*Bs*DnaA) interacts with SirA, the sporulation-related protein (Rahn-Lee et al., 2011). However, the binding partner proteins can vary among DnaA orthologs, and replication initiator from one bacterium can interact with different partners compared to other orthologs, e.g., interaction of *Thermoanerobacter tengcongensis* DnaA with NusG protein, is not observed for *Bs*DnaA (Liu et al., 2008). The second domain, forming a flexible linker, although it is not essential (Messer et al., 1999; Nozaki and Ogawa, 2008), was proposed to be involved in optimal helicase DnaB recruitment (Molt et al., 2009). The domain II, links domain I with domain III, which contains a common core structure of AAA+ proteins family members (Neuwald et al., 1999). Recent data showed that residues within this domain are engaged in interaction of DnaA (*Tm*DnaA, *Ec*DnaA, *Aa*DnaA) with single-stranded DNA (ssDNA; Ozaki et al., 2008; Duderstadt et al., 2011). At the C-terminus of DnaA, domain IV (DNA Binding Domain, DBD) can be distinguished, which is responsible, via a helix-turn-helix motif (HTH), for interaction with double-stranded DNA (dsDNA) containing specific motifs named DnaA-boxes (Roth and Messer, 1995; Fujikawa et al., 2003). Interaction with these sequences is the very first step of the replication initiation process.

**Figure 3:**
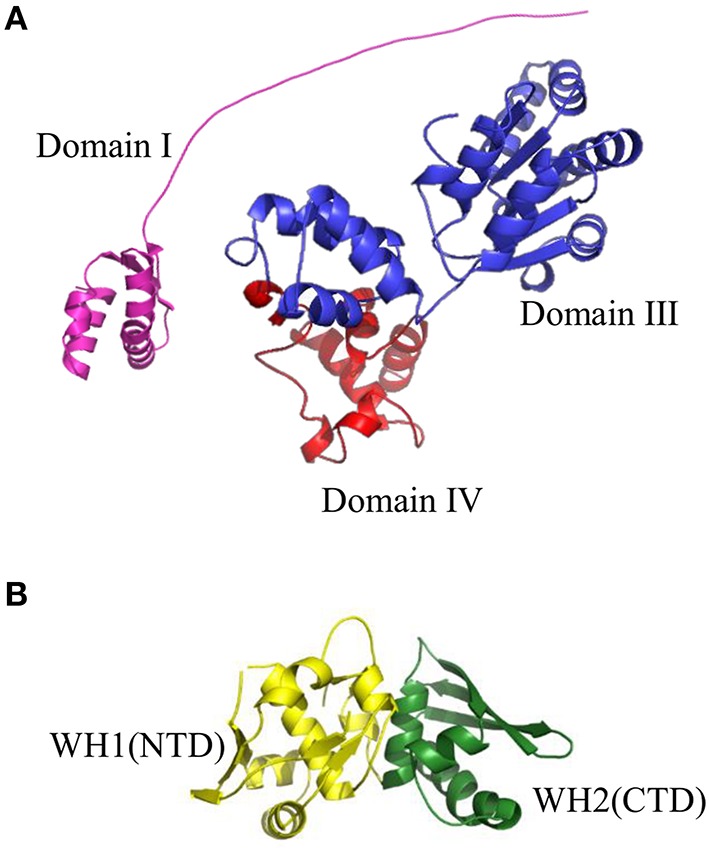
**Structures of replication initiation proteins: (A) bacterial DnaA protein and (B) RepE protein from plasmid F. (A)** Crystal structure of Domain I (shown in violet) of *Ec*DnaA protein was obtained from the PDB database (2E0G). Domain III (shown in blue) and Domain IV (shown in red) were modeled using SWISS-MODEL server (http://swissmodel.expasy.org) basing on crystal structure of Domain IV obtained from PDB database (1J1V). The presented structure of *Ec*DnaA does not include Domain II. **(B)** Crystal structure of RepE protein, comprising Winged Helix domain 1 (WH1), and Winged Helix domain 2 (WH2) (shown in yellow and green, respectively), were obtained from the PDB database (1REP).

In bacterial chromosome *origin*, regions that are composed of a variable number of DnaA-boxes, can be identified (Ozaki and Katayama, 2009; Rajewska et al., 2012; Wolanski et al., 2014; Leonard and Grimwade, 2015). In the *origin* of *E. coli* chromosome (*oriC*), five 9-bp in length DnaA-boxes (R1–R5) were originally identified (Fuller et al., 1984; Matsui et al., 1985); in contrast, the *origin* of *Caulobacter crescentus* chromosome (*Cori*) possesses only two DnaA-boxes (named G-boxes; Shaheen et al., 2009). The studies with the use of *in vivo* and *in vitro* dimethylsulphate (DMS) footprinting as well as DNase I footprinting method showed that other, non–R DnaA binding sites are present in *oriC*, i.e., I (Grimwade et al., 2000; McGarry et al., 2004), C (Rozgaja et al., 2011), and τ sites (Kawakami et al., 2005). Such non-canonical sequences recognized by bacterial initiator were also found in *oriC* of *C. crescentus* (termed W-boxes; Taylor et al., 2011). The affinity of DnaA binding to R-boxes and non-R DnaA binding sites is different. Interestingly, binding of inititor to the DnaA-boxes in *Cori* of *C. crescentus*, both G-boxes and W-boxes, is lower compared to DnaA binding to the R-boxes in *oriC* of *E. coli* (Taylor et al., 2011), which might be characteristic for bacteria with a complex regulation of development. The DnaA binding sites, bound by initiator with affinity comparable only to interaction between DnaA and weak DnaA-boxes in *E. coli oriC*, were found in the *origin* of *H. pylori* (Zawilak-Pawlik et al., 2007; Charbon and Løbner-Olesen, 2011). In *E. coli oriC* three (named R1, R2, and R4) out of five DnaA-boxes are the widely separated, high affinity DnaA-boxes. They were found to be almost constantly bound by *Ec*DnaA protein (Samitt et al., 1989; Nievera et al., 2006). The occupancy of only these three sites is insufficient for spontaneous *origin* opening and it was proposed that interaction of *Ec*DnaA protein at high affinity binding sites may regulate conformation of the *origin* DNA (Kaur et al., 2014). Between the peripheral R1 and R4 sites, there are two arrays of low affinity binding sites, τ1 R5 τ2 I1 I2 and C3 C2 I3 C1, separated by one of high affinity—R2 (Rozgaja et al., 2011). *Ec*DnaA molecules bound to the high affinity DnaA-boxes, termed bacterial Origin Recognition Complex (bORC), act as anchors and are required to assist in occupying weak sites by the *Ec*DnaA protomers (Rozgaja et al., 2011; Kaur et al., 2014), and formation of replication-active pre-replication complex (pre-RC; Figure 2, Stage II). The binding affinity to particular sequences and replication activity of *Ec*DnaA protein depend on nucleotide-bound state of protein. Although ADP-*Ec*DnaA binds the high affinity DnaA-boxes and also R5 and C1 low affinity ones, the ATP-*Ec*DnaA form is thought to be the replication-active one (Sekimizu et al., 1987; Leonard and Grimwade, 2011). ATP-*Ec*DnaA form of initiator binds efficiently both high and low affinity binding sites (McGarry et al., 2004; Kawakami et al., 2005). Based on molecular docking, binding of ATP, instead of ADP, is presumed to cause changes in the *Ec*DnaA protein conformation, thus leading to the formation of large oligomeric complex within the *origin* region (Saxena et al., 2015). The crystallographic data, when nonhydrolyzable ATP analog AMP-PCP was used, showed the formation of open-ended, right-handed helical filament of *Aa*DnaA (Erzberger et al., 2006). Based on biochemical and genetic approaches it was found that there is an interaction between domain III (AAA + domain) of one DnaA (*Ec*DnaA or *Aa*DnaA) molecule and domain IV (DBD domain) of partner subunit (Duderstadt et al., 2010). It was proposed that during pORC and pre-RC complexes formation of the DBD domain is extended and the HTH motif is exposed, which results in the efficient binding of high and low affinity binding sites (Duderstadt et al., 2010). Occupation of the *Ec*DnaA binding sites was shown to be sequential and polarized and DnaA protomers are released preferentially from the peripheral high affinity R1 and R4 boxes, through arrays of low affinity binding sites to the middle high affinity one—R2 (Rozgaja et al., 2011). The formation of DnaA oligomer within the *oriC* results in DNA destabilization in the DUE region (Speck and Messer, 2001; McGarry et al., 2004; Leonard and Grimwade, 2005, 2011; Duderstadt et al., 2010). Although two arrays of low affinity binding sites separated by high affinity sequences are occupied by *Ec*DnaA protomers for efficient double-stranded DNA opening, binding of *Ec*DnaA to a part of *origin* (containing only R1 high affinity box and τ1 R5 τ2 I1 I2 low affinity binding sites array) was shown to be active in DUE unwinding (Ozaki and Katayama, 2012). It was proposed that distinct DnaA multimers are formed on the left half (containing binding sites from R1 to I2) and the right half (containing binding sites from R2 to R4) of *oriC* (Ozaki and Katayama, 2012; Ozaki et al., 2012a).

The DUE melting is the consequence of DnaA binding to arrays of DnaA-boxes (Figures 1A, 2, Stage II). The location of particular binding sites suggests that DnaA, bound to sequences of the high affinity DnaA-boxes (R1, R2, R4), could cause the bending of DNA molecule via interaction through domain I of already bound three protomers (Rozgaja et al., 2011; Kaur et al., 2014; Leonard and Grimwade, 2015). The model of constrained loop formed by *Ec*DnaA bound to the high affinity binding sites was proposed (Kaur et al., 2014). The bending of *oriC* containing DNA molecule is supported by accessory histone-like proteins HU and integration host factor (IHF). A binding site for IHF was found within the *oriC* region (Polaczek, 1990) and it was shown that IHF can enhance the unwinding of DNA by DnaA (Hwang and Kornberg, 1992; Ryan et al., 2002). It was demonstrated that HU has the same effect on DUE destabilization (Hwang and Kornberg, 1992), although its mechanism of action is different (Ryan et al., 2002). Data obtained with ELISA (Enzyme Linked Immunosorbent Assay) showed that HU interacts with domain I of *Ec*DnaA, which was proposed as an interaction which stabilizes the DnaA oligomer (Chodavarapu et al., 2008a). The Fis protein, identified originally as factor for inversion stimulation in site-specific DNA recombination, was also shown to have an influence on DNA unwinding (Wold et al., 1996). Specific binding sites for Fis were identified in *oriC* (Gille et al., 1991). Although Fis, in contrast to IHF, negatively regulates DNA replication initiation, when the *origin* lacks some DnaA binding sites resulting in altered non-functional conformation of *origin*, both Fis and IHF can work together to correct these alterations (Kaur et al., 2014). This joint action is achieved by inducing bends in *oriC* and establishing functional *origin* conformation (Kaur et al., 2014).

The formation of DnaA oligomer with synergistic action of architectural proteins can introduce torsional strain into DUE, facilitating the melting of the double-stranded DNA helix. The binding of DnaA to DUE region was also thought to introduce DNA melting, and ATP-DnaA-boxes were distinguished within the *oriC* DUE sequence (Speck and Messer, 2001). Recent studies showed direct binding of *Ec*DnaA and *Aa*DnaA protein to formed single-stranded DNA within the DUE (Ozaki et al., 2008; Duderstadt et al., 2010, 2011; Cheng et al., 2015). Studies with DnaA mutants (Ozaki et al., 2008; Duderstadt et al., 2010), as well as crystallography (Duderstadt et al., 2011), showed that this interaction occurs through residues located within the AAA+ domain III of bacterial initiator. The *Aa*DnaA protomers form a helical filament on ssDNA (Duderstadt et al., 2011), however, it differs from the filament formed on the dsDNA (Erzberger et al., 2006; Duderstadt et al., 2010). It was proposed that protomers in this oligomer are more compact when compared to the extended DnaA molecules in dsDNA-DnaA complex (Duderstadt et al., 2010, 2011). The binding of ssDNA concerns just one T-rich strand of DUE and depends on sequence of 13-nucleotide sequences, which can be distinguished within the DUE. In *oriC* three 13-mers are present (Bramhill and Kornberg, 1988a) and the binding of *Ec*DnaA occurs at least two 13-mers. *Ec*DnaA does not form a complex with ssDNA containing just one 13-mer (Ozaki et al., 2008). Formation of this nucleoprotein complex is achieved only by ATP-DnaA protein (Ozaki et al., 2008) and one *Aa*DnaA protomer binds three nucleotides of ssDNA (Duderstadt et al., 2011; Cheng et al., 2015). Studies with the use of single-molecule fluorescence assays showed that the formation of this nucleoprotein complex is highly dynamic and that *Aa*DnaA molecules assemble on ssDNA in the 3′ to 5′ direction (Cheng et al., 2015). The presence of dsDNA region containing DnaA-boxes, adjacent to ssDNA DUE, stabilizes the DnaA (*Ec*DnaA and *Aa*DnaA) filament on ssDNA (Ozaki and Katayama, 2012; Cheng et al., 2015). Recently published data revealed presence of a new origin element, termed DnaA-trio, composed of repeated trinucleotide motif that stabilizes DnaA filaments on the ssDNA (Richardson et al., 2016). What is important, binding single strand of DUE region is required for *origin* activity (Ozaki et al., 2008, 2012a,b; Duderstadt et al., 2011).

### Origin recognition and open complex formation by plasmid initiator at *origin* of iteron plasmids

Similarly as during bacterial chromosome replication, the first step in open complex formation in many theta-replicating plasmids, especially in iteron-containing plasmids, is the binding of plasmid replication initiator, Rep protein, to specific sequences within *origin* region (Figure 2, Stage I). Rep proteins are structurally different from bacterial DnaA protein and consist of winged-helix (WH) domains (Figure 3B, Komori et al., 1999; Díaz-López et al., 2003; Sharma et al., 2004; Swan et al., 2006; Nakamura et al., 2007a,b; Pierechod et al., 2009). The crystal structures of nucleoprotein complexes of π protein from plasmid R6K (Swan et al., 2006), RepE protein from plasmid F (Komori et al., 1999; Nakamura et al., 2007b), and a DNA binding domain of Rep protein from ColE2–P9 plasmid (Itou et al., 2015) as well as N-terminal domain of RepA protein from plasmid pPS10 (Giraldo et al., 2003) were obtained. Furthermore, homological models for plasmid Rep proteins: RepA from P1 (Sharma et al., 2004), RepA from pSC101 (Sharma et al., 2004), and TrfA from RK2 (Pierechod et al., 2009) were shown. Plasmid Reps are composed of two WH domains, of which one is responsible for oligomerization and the role of a second one is the protein's interaction with DNA (Giraldo et al., 1998; Nakamura et al., 2004; Pierechod et al., 2009). Plasmid replication initiators are present as dimers in solution, however, an exception is known i.e., RepE protein from pAMβ1 plasmid is present as a monomer (Le Chatelier et al., 2001). Although the Rep dimers interact with DNA (Filutowicz et al., 1985; Ingmer et al., 1995; Komori et al., 1999), they are replication-active in the monomeric form (Kawasaki et al., 1990; Wickner et al., 1992; Sozhamannan and Chattoraj, 1993; Konieczny and Helinski, 1997). Conformational activation of plasmid replication initiators is carried out by chaperon proteins (Kawasaki et al., 1990; Wickner et al., 1992, 1994; Sozhamannan and Chattoraj, 1993; Konieczny and Helinski, 1997). In contrast to bacterial replication initiator DnaA, the domain responsible for binding of nucleotide was not identified in Reps' structures. There is also no evidence showing if Rep proteins can form helical filaments on DNA similar to that formed by the *Aa*DnaA protein. For some Reps, e.g., TrfA protein from RK2 plasmid, two forms of protein, different in length, occur: the shorter 33 kDa (TrfA-33) and longer 44 kDa (TrfA-44). There are different requirements for each particular form depending on the host bacterium. In *E. coli* both forms of TrfA can initiate the plasmid replication, whereas in *Pseudomonas aeruginosa* only the longer form is active (Caspi et al., 2001; Jiang et al., 2003; Konieczny, 2003; Yano et al., 2013, 2016).

During plasmid replication initiation, monomers of Reps bind to specific repeated sequences, named iterons, present within *origin* region (Figures 1B, 2, Stage I). The number of iterons varies among plasmid *origins*, from two iterons in plasmids ColE2 and ColE3 (Yasueda et al., 1989), three iterons in pSC101 (Churchward et al., 1984), and some plasmids from IncQ incompatibility group (Loftie-Eaton and Rawlings, 2012), four iterons in *origin* of plasmid F and pPS10, up to five (*origin* of plasmids RK2 and P1) or even seven such sequences in *oriƔ* of plasmid R6K (Rajewska et al., 2012). Iterons are short sequences, in length ranging from 17-bp in RK2 plasmid (Stalker et al., 1981), 19-bp in plasmids F (Murotsu et al., 1981), and P1 (Abeles et al., 1984), to 22-bp in R6K (Filutowicz et al., 1987), and pPS10 (Nieto et al., 1992). But in some plasmids the iteron sequences which are present in one *origin* can differ in length and apart from short sequences, significantly longer iterons [up to even 76-bp in plasmid R478 from IncHI2 incompatibility group (Page et al., 2001)] can be present. The binding of Rep proteins to iterons is sequence-specific and mutations in these motifs disrupt binding of plasmid initiation protein. Changes in a sequence of iterons abolished binding of π protein within the *oriƔ* of plasmid R6K and thus replication activity *in vivo* (McEachern et al., 1985). Negative effects on replication was also observed for mutants in a sequence of P1 plasmid iterons (Brendler et al., 1997). The sequences separating particular iterons are also important for Rep nucleoprotein complexes formation and proper replication activity of *origin*. It was shown in case of the RK2 plasmid that *in vitro* the TrfA protein has a high preference for binding to DNA containing at least two out of five binding sites, when compared to the formation of nucleoprotein complex with DNA containing just one iteron (Perri et al., 1991). The requirement for the presence of more than just one iteron sequence for TrfA binding was also shown *in vivo* (Perri and Helinski, 1993). Rep proteins bind to iterons in a cooperative manner (Perri and Helinski, 1993; Xia et al., 1993; Bowers et al., 2007) and the cooperativity of binding depends on the spatial location of iterons, since separation of two iterons by a half helical turn abolished cooperativity (Bowers et al., 2007). These results suggest the possibility of formation of higher order nucleoprotein structure on plasmid iterons bound by Reps. It was shown that WH domains of Reps contact three nucleotides in DNA. In π protein from R6K plasmid, WH1 domain contacts wGwnCnT motif, and WH2 domian contacts GAG sequence (Swan et al., 2006). Similarly, the WH2 domain of RepE monomer also contacts three nucleotides of top (GTG sequence) and three nucleotide of bottom strand (GtCA sequence) of double-stranded molecule containing iteron sequence (Nakamura et al., 2007b). However, unlike for the bacterial DnaA protein, to date there are no evidence showing that strong and weak binding sites for Reps are present within plasmid *origins*. There were just predictions of potential binding sites, other than iterons, for π protein in R6K plasmid and suggestions on potential role of such sites (Rakowski and Filutowicz, 2013). Certainly like DnaA, Rep proteins can bind within single-stranded region of melted DUE, and this binding is sequence-specific, since binding concerns a particular strand. Nucleoprotein complexes formation with the ssDNA DUE was detected for TrfA (bound with A-rich strand) and RepE (bound with T-rich strand) proteins (Wegrzyn et al., 2014). Within the DUE of plasmid *origins*, repeated sequence, similar to 13-mers distinguishable in *oriC*, can be found. There are four 13-nucleotide sequences in plasmid RK2 DUE region (Doran et al., 1998) and all of them are required for TrfA-ssDNA DUE complex formation. Lack of even one 13-mer hinders plasmid replication (Wegrzyn et al., 2014). Also, even a point mutation within this region affects plasmid replication since the lack of DUE melting was observed for some of the changed sequences (Kowalczyk et al., 2005; Rajewska et al., 2008).

The Rep protein encoded by plasmids, can be accompanied by host DnaA initiator during open complex formation and DUE melting within a plasmid *origin* (Figure 2, Stage II). DnaA binding sites have been found in replication *origin* of many plasmids including plasmids P1 (Abeles et al., 1984, 1990; Abeles, 1986), F (Kline et al., 1986; Murakami et al., 1987; Kawasaki et al., 1996), RK2 (Doran et al., 1998; Caspi et al., 2000), pSC101 (Sutton and Kaguni, 1995). The number of DnaA-box sequences differs among plasmid *origins*, the position and orientation of these binding sites are as important as position and orientation of the iterons (Doran et al., 1998, 1999). The inversion of one out of four DnaA boxes in *origin* of RK2 plasmid abolished plasmid DNA replication, despite the fact that three remaining DnaA boxes were bound by the host initiator (Doran et al., 1999). Although the DnaA protein is not required for replication initiation for some plasmids, e.g., R1, binding of DnaA increased the plasmid replication efficiency (Bernander et al., 1991, 1992) and mutations within a binding site for DnaA decreased the R1 plasmid replication (Ortega-Jiménez et al., 1992). In bacteria, ATP-DnaA form is essential for chromosomal DNA replication (Sekimizu et al., 1987; Leonard and Grimwade, 2005, 2011). Interestingly, studies with ATP-binding mutant of DnaA, which was inactive in *oriC* replication, showed that bacterial initiator lacking an ability to bind a nucleotide was effective in open complex formation within plasmid R6K *oriƔ* (Lu et al., 1998). Also in the presence of ATPS, a non-hydrolyzable analog of ATP, the pattern of bands in KMnO_4_ footprinting assay with DnaA and TrfA proteins and plasmid RK2 DNA showed no significant differences, when compared to opening reaction containing ATP (Konieczny et al., 1997). Thus, the DnaA is suspected to play a different role in plasmid replication initiation, compared to its role in chromosome replication. A direct interaction between plasmid and host replication initiators was shown (Lu et al., 1998; Maestro et al., 2003) and the interaction was detected in the N-terminus of π (between 1 and 116 aa) protein of R6K plasmid (Lu et al., 1998) and RepA protein of pSC101 (Sharma et al., 2001) and domain I and IV of host initiator (Sharma et al., 2001). The mutations in RepA protein from pPS10 plasmid were introduced, which enhanced the interaction of RepA with DnaA protein and resulted in changes in host range of pPS10 plasmid (Maestro et al., 2003).

Similarly to bacterial chromosome replication initiation, the binding of DnaA protein to DnaA-boxes within plasmid *origins* can be enhanced by the presence of architectural proteins IHF, and HU (Shah et al., 1995; Fekete et al., 2006). The binding of IHF to its binding site in *oriƔ* region significantly enhanced binding of bacterial DnaA to R6K plasmid *origin* (Lu et al., 1998). In pSC101 plasmid binding IHF to its cognate binding site is required for plasmid replication initiation and mutations within this sequence disrupts plasmid replication (Stenzel et al., 1987). For plasmid P1 the binding of IHF to its site, located downstream of one out of two arrays of DnaA-boxes (the second array is located upstream of DUE) is required only when the nearby DUE array of DnaA-boxes is not active and the second DnaA-boxes array serves as a secondary origin compensating the function of the first one (Fekete et al., 2006). The P1-mini derivative was just slightly unstable in IHF *E. coli* mutant (Ogura et al., 1990). The mutations in gene for IHF protein did not affect plasmids F (Ogura et al., 1990) and RK2 (Shah et al., 1995) replication. In contrast, the lack of HU protein *in vitro* results in significant decrease in mini-F plasmid DNA synthesis (Zzaman et al., 2004) and *in vivo* KMnO_4_ reactivity of P1 plasmid *origin* (Park et al., 1998) as well as abolishment of plasmid F replication *in vivo* (Ogura et al., 1990). During plasmid RK2 replication initiation, HU could functionally replace DnaA protein, although it could not enhance DUE melting as efficiently as DnaA (Konieczny et al., 1997). It was proposed that one of the DnaA functions could be the stabilization of *origin* melting induced by Rep protein. The other DnaA role during replication initiation is its function in helicase loading. Interestingly, for some plasmids, e.g., RK2, DnaA assists Rep during plasmid replication initiation only in particular hosts, while in others DnaA is dispensable [DnaA *P. aeruginosa* is dispensable for RK2 plasmid replication initiation, but required in *E. coli* (Caspi et al., 2001; Konieczny, 2003)].

## Helicase loading, activation, and DNA unwinding

In bacteria the loading of DnaB helicase onto ssDNA of DUE is achieved by the action of replication initiation protein, DnaA, as well as the helicase loading factor, DnaC protein (Figure 2, Stage III). DnaB helicase is a two-tiered ring-shaped hexamer (Bailey et al., 2007b; Wang et al., 2008; Lo et al., 2009). Each monomer consists of N-terminal and C-terminal domain connected via linker helix (LH) region (Miron et al., 1992; Ingmer and Cohen, 1993; Komori et al., 1999). The N-terminal domain of helicase's monomers were shown to interact with ssDNA (observed in a crystal structure of *Geobacillus kaustophilus* helicase in a complex with ssDNA; Lo et al., 2009) which stabilizes the hexameric structure of DnaB (Biswas et al., 1994). The C-terminal domain, that contains RecA-like fold, is responsible for ATP binding and hydrolysis, interaction with DNA (Bailey et al., 2007a), and binding of DnaC loader factor (Lu et al., 1996). The helicase is positioned onto the ssDNA DUE in a single orientation with respect to the polarity of the sugar-phosphate backbone of DNA and the nucleic acid, bound primarily to one DnaB monomer (Jezewska et al., 1998a,b), passes through the cross-channel of helicase hexamer (Jezewska et al., 1998a). The hexamer of DnaB, when no ATP hydrolysis occurs, is bound to 20 (±3) nucleotides (Jezewska et al., 1996).

The binding of nucleotide as well as particular partner protein and DNA promotes helicase to adopt specific conformation. The X-ray crystal structure of *A. aeolicus* helicase revealed large conformational rearrangements, observed in N-terminal domain and the presence of at least two highly-distinct conformations: widened with broad central channel and a highly-constricted with a narrow pore (Strycharska et al., 2013). These conformations were also observed for *E. coli* DnaB, when analyzed in solution with the use of small-angle X-ray scattering (SAXS; Strycharska et al., 2013). Structural analysis with the use of negative-stain electron microscopy (EM) and SAXS of DnaB protein in complex with its loader, DnaC, showed that the hexamer of helicase interacts with helical arrangement of six DnaC monomers (Kobori and Kornberg, 1982; Arias-Palomo et al., 2013). However, it was argued that the active form of the DnaB-DnaC complex exists in 6:3 stoichiometry, which was studied by quantitative analysis of pre-priming complex (Makowska-Grzyska and Kaguni, 2010). Furthermore, the imbalance in level of DnaB and DnaC was shown to impair DNA replication (Allen and Kornberg, 1991; Brüning et al., 2016).

The concept of DnaC as a protein that loads DnaB helicase onto ssDNA of DUE, has been early established (Wickner and Hurwitz, 1975; Funnell et al., 1987; Bell and Kaguni, 2013). To further explain its exact function, the following models have been proposed: (1) DnaC breaks the helicase ring (Davey and O'Donnell, 2003; Arias-Palomo et al., 2013), (2) DnaC traps DnaB helicase as an open ring (Chodavarapu et al., 2016). Those hypotheses were tested by the SAXS method and deuterium exchange coupled to mass spectrometry, respectively. The ATPase activity of DnaC, a member of AAA+ proteins family, is not required for helicase hexamer opening and its loading by DnaC, hence the DnaB-binding domain of loader is sufficient for this process (Arias-Palomo et al., 2013). Yet the ATP hydrolysis by DnaC was proposed to occur during DnaB helicase activation, which results in DNA unwinding (Felczak et al., 2016).

Regarding the DnaC key contribution to helicase loading and activation in *E. coli*, it is particularly interesting to discuss replicons that are independent of helicase loader. The helicase loaders were identified only in few species and it is possible that in some bacteria the yet unidentified helicase loaders are present. The lack of DnaC orthologs can also arise from ability of self-loading by helicase (Costa et al., 2013) or it is possible that another protein of already assigned role, substitutes the DnaC function. Those hypotheses can be supported by complementation of *dnaC* temperature-sensitive mutant of *E. coli* by helicase from *H. pylori* (Soni et al., 2003). The dispensability for helicase loader was also shown during RK2 plasmid replication in *Pseudomonas* species (Jiang et al., 2003). In *Pseudomonas* sp. the helicase loading at plasmid RK2 *origin* is performed by the longer form of plasmid Rep protein, TrfA-44, which interacts with *Pseudomonas* helicase (Caspi et al., 2001; Jiang et al., 2003; Zhong et al., 2003). The shorter form of this plasmid initiator, TrfA-33, is not sufficient for helicase loading in *P. aeruginosa*. In *Pseudomonas putida* TrfA-33 can load helicase but only in the presence of DnaA (Caspi et al., 2001; Jiang et al., 2003). On the contrary, the DnaC helicase loader, together with DnaA, and Rep protein (either short or long form), is absolutely required for helicase loading at plasmid RK2 *origin* in *E. coli* (Caspi et al., 2001). It was shown that via interaction of DnaA with DnaBC, the helicase is first localized in DnaA-boxes and then via DnaA-DnaB and Rep-DnaB interactions translocated to ssDNA DUE (Pacek et al., 2001; Rajewska et al., 2008). Probably the Rep-DnaA interaction is also important in these processes. Apart from the proper protein-protein interaction, an efficiency of helicase translocation from DnaA-box position to DUE depends on the sequence of DUE region. It was shown via electron microscopy and *in vitro* experiments that even point mutations within the DUE of RK2 plasmid *origin* results in a decrease in helicase translocation and thus helicase DNA unwinding activity (Rajewska et al., 2008).

It was proposed that, upon DnaB-DnaC binding to ssDNA, DnaC dissociates, thus allowing DnaB to unwind double helix, and further to bind DnaG primase (Wahle et al., 1989, Figure 2, Stage IV). However, Makowska-Grzyska and Kaguni demonstrated, by performing molecular filtration of pre-priming complex at *E. coli oriC*, that the DnaG primase binds DnaB, synthesizes primer and in consequence, induces the release of DnaC from DnaB (Makowska-Grzyska and Kaguni, 2010). In *E. coli*, in further steps DnaG primase is associated with DnaB helicase and synthesizes primers on lagging strand (McHenry, 2011). Plasmid ColE2-P9 does not require DnaG primase in replication initiation (Takechi et al., 1995). Itoh group demonstrated that ColE2 *origin* and Rep protein as well as *E. coli* host DNA Polymerase I and SSB are sufficient for *in vitro* DNA synthesis (Itoh and Horii, 1989). Further studies revealed that the ColE2-Rep protein has joined functions, i.e., replication initiator and plasmid-specific primase (Takechi and Itoh, 1995).

Once activated, DnaB unwinds one nucleotide per one catalytic step in ATP-dependent manner (Lohman and Bjornson, 1996, Figure 2, Stage IV). It was shown that at 25°C the DnaB unwinds around 291 bp per second (Galletto et al., 2004) and it moves from 5′ to 3′ direction along the ssDNA (LeBowitz and McMacken, 1986). Because the replication of bacterial chromosome is bidirectional two helicases are loaded: one is loaded by DnaC on the top strand invaded by DnaA molecules and the other on the bottom strand. It was proposed that the helicase delivery to ssDNA DUE bottom A-rich strand occurs by direct interaction between DnaB and DnaA proteins (Mott et al., 2008; Soultanas, 2012). The Phe-46 of DnaA was shown to be important for this interaction (Keyamura et al., 2009). The order of helicase loading to a particular strand of DUE is not random but defined; first helicase is loaded onto the bottom/lower strand then the second onto the top/upper one (Weigel and Seitz, 2002). Such order of helicase loading probably supplies head-to-head orientation of unwound region of *oriC* and prevents back-to-back loading of the helicase. The basal level of DnaB activity in *oriC* is achieved when DnaA forms an oligomer in ssDNA DUE and dsDNA containing DnaA-boxes from R1 to I2 (called DAR-DF and DAR-LL). For the full activity of helicase the formation of DnaA filament on other DnaA-boxes (from R2 to R4; called DAR-RL and DAR-RE) is needed (Ozaki and Katayama, 2012).

The interaction between plasmid initiator Rep and helicase is an important factor for helicase activity on plasmid *origin* (Figure 2, Stage IV). It was shown for *E. coli* F plasmid that its initiator, RepE protein, cannot form a stable complex with *Pseudomonas* helicase and thus it does not replicate efficiently in *Pseudomonas* cells (Zhong et al., 2005). Interaction between plasmid Rep and host DnaB was also detected via ELISA and protein affinity chromatography for π protein of R6K (Ratnakar et al., 1996) and mutations within π were identified which decreased helicase binding and resulted in impaired plasmid DNA replication (Swan et al., 2006). A similar effect was observed for mutants of RepA protein form plasmid pSC101, invalid in the interaction with helicase (Datta et al., 1999). Although the Rep-DnaB interaction is required for helicase loading, the right balance in the strength of the interaction must be maintained. It was shown that too tight binding of Rep to DnaB is undesirable and the mutations within Rep, acquired by adaptation under antibiotic selection that decreased binding to helicase, result in the decrease in fitness cost and increase in plasmid copy number (Yano et al., 2016).

## Replisome assembly

Once DnaB helicase is loaded, DNA is unwound and primer is synthesized, the contribution of replication initiators becomes more enigmatic. Subsequent stages of DNA replication require building the replisome, i.e., the multiprotein replication machinery that synthesizes DNA (O'Donnell et al., 2013, Figure 2, Stage V). The replisome in bacteria is composed of DnaB helicase, DnaG primase, single-stranded DNA binding protein (SSB), and the holoenzyme of DNA Polymerase III (hPol III) (divided in three subcomplexes: Pol III core, clamp loader and β-clamp; O'Donnell, 2006). Reyes-Lamothe et al., suggested that both DnaA replication initiator and DnaC helicase loader are crucial for replisome assembly in *E. coli* (Reyes-Lamothe et al., 2008). This conclusion was drawn from studies that tracked the replisome components in living cells during the stages of DNA replication. However, it does not exclude the possibility that the role of replication initiator is limited to DUE destabilization and helicase loading, hence, indirect effects may be observed. Most studies regarding the mechanism of replisome assembly are performed using simplified experimental setup, e.g., primed ssDNA and replisome components, where replication initiators are omitted (Yuzhakov et al., 1996, 1999; Downey and McHenry, 2010; Cho et al., 2014).

### Clamp loader and its activity

Following the primer synthesis, clamp loader complex loads the ring-shaped β-clamp (discussed below in details), that encircles dsDNA, tethers DNA polymerase, and slides along dsDNA, thus significantly increasing speed (up to 100-fold), and processivity (up to 5000-fold) of DNA replication (Kelch et al., 2012). The *E. coli* clamp loader is composed of γ, τ, δ, δ', χ, and ψ subunits, albeit only γ, δ, δ' are crucial for β-clamp loading (reviewed in details in Kelch, 2016). The γ subunit is a truncated version of τ subunit, encoded by *dnaX* gene, and arises from translational frameshift (Flower and McHenry, 1990). Both γ and τ subunits have AAA^+^ domain, however, γ subunit lacks τ subunit domain responsible for DnaB helicase and Pol III core binding (Tsuchihashi and Kornberg, 1989; O'Donnell and Studwell, 1990; Flowers et al., 2003). Before clamp loader binds DNA, it adopts appropriate, ATP-driven conformational state that increases its affinity for the β-clamp (Podobnik et al., 2003). It is under debate whether the ring structure of β-clamp is actively opened or captured in open conformation. The T4 bacteriophage trimeric clamp is the least stable sliding clamp and it was found to dissociate from DNA by monomerization, thus no force in opening of the ring is required (Soumillion et al., 1998). The dimeric clamps (bacterial, e.g., *E. coli*) are regarded as stable, hence more active ring-opening mechanism is expected to be in demand. On the basis of a crystal structure of single subunit of *E. coli* clamp loader (namely δ subunit) in complex with β-clamp, it was proposed that δ subunit is a molecular wrench, that induces rearrangements of β-clamp at dimerization interface, albeit without ATP hydrolysis (Jeruzalmi et al., 2001). With the use of real-time fluorescence-based clamp binding and opening assays, it was shown that clamp loader binds closed β-clamp in solution, prior to β-clamp opening (Paschall et al., 2011). Yet, the deuterium exchange coupled to Mass Spectrometry experiments revealed that most sliding clamps are dynamic at their monomers' interfaces (Fang et al., 2011, 2014). Therefore, it is also probable that β-clamp is trapped in an open conformation by clamp loader.

The crystal structure of clamp loader complex was solved from T4 bacteriophage (Kelch et al., 2011), *E. coli* (Simonetta et al., 2009), and its eukaryotic homolog, Replication Factor C (RFC), from *Saccharomyces cerevisiae* (Bowman et al., 2004). Each of the clamp loader complex reveals pentameric structure. Since AAA + ATPases usually adopt circular hexamers, it was proposed that sixth subunit was lost during the evolution (Indiani and O'Donnell, 2006). Indeed, the gap between the first and fifth clamp loader subunits is favorable, because it provides the mechanism of specific accommodation of the primer-template junction structure (Kelch, 2016). It was suggested that clamp loader recognizes minor groove and thus it binds at the 3′ primer-template junction specifically. However, the crystal structure of the clamp loader:DNA complex revealed that clamp loader contacts template DNA exclusively (Bowman et al., 2005; Simonetta et al., 2009). Despite the fact that the DNA synthesis may be initiated only from 3′ OH primer end, the clamp loader can assemble *in vitro* at either 3′ or 5′ primer terminus forming a stable complex (Park and O'Donnell, 2009). While clamp loader binds only DNA template, β-clamp interacts with both RNA primer, and DNA template within the RNA-DNA hybrid and it was shown that the β-clamp distinguishes between the 5′ and 3′ primer end (Park and O'Donnell, 2009). Consistently, it was demonstrated that SSB hampers clamp loading on the 5′end of primer (Hayner et al., 2014). The ATPase activity of the clamp loader is lower when it is assembled at the 5′ terminus, comparing to the ATPase activity of clamp loader located at 3′ terminus (Park and O'Donnell, 2009). ATP hydrolysis triggers β-clamp closing on DNA and the release of clamp loader from β-clamp:DNA nucleoprotein complex (Pietroni and von Hippel, 2008). Thereby, the 3′ primed end loading preference also arises from the higher rate of clamp closure and clamp loader dissociation (Park and O'Donnell, 2009). The β-clamp must be closed in the ATP hydrolysis-dependent manner, to release clamp loader (Hayner et al., 2014). Clamp loader must free the β-clamp to allow the Pol III core to bind, since they accommodate the same binding site within the β-clamp, namely the hydrophobic cleft.

### β-clamp—hub for protein interactions

β-clamp crystal structures were obtained from various organisms i.e., *E. coli* (Oakley et al., 2003; Burnouf et al., 2004), *P. aeruginosa* (Wolff et al., 2014), *Streptococcus pyogenes* (Argiriadi et al., 2006), *M. tuberculosis* (Gui et al., 2011; Kukshal et al., 2012; Wolff et al., 2014), *B. subtillis* (Wolff et al., 2014), *T. maritima* (structure 1VPK), *Eubacterium rectale* (structure 3T0P)*, Streptococcus pneumoniae* (Argiriadi et al., 2006). The crystal structures of β-clamp homologs—Proliferating Cell Nuclear Antigen (PCNA)—are also available from *Eukaryotes* and *Archea* (to name a few: *Homo sapiens* (Punchihewa et al., 2012), *S. cerevisiae* (Krishna et al., 1994), *Sulfolobus solfaraticus* (Williams et al., 2006). All of them adopt ring shaped homodimer (e.g., *E. coli*) or homotrimer (human PCNA, *Pyrococcus furiosus* PCNA), albeit the exception is PCNA of *Archea, S. solfataricus*, which exists as a heterotrimer (Dionne et al., 2003). β-clamp monomers bind in a head to tail manner (Kelman and O'Donnell, 1995). The β-clamp and PCNA structure is conserved among all kingdoms of life, in contrast to amino acid sequence (Jeruzalmi et al., 2001). However, the amino acid sequence of region termed hydrophobic cleft was found to be highly conserved (Jeruzalmi et al., 2001). The hydrophobic cleft is a site for interaction with β-clamp binding partners (Jeruzalmi et al., 2001). β-clamp forms a protein interaction hub and serves as a platform for multiple protein interactions crucial in various cellular processes, i.e., DNA elongation in every living organism (Hedglin et al., 2013), regulation of DNA replication in *E. coli, B. subtilis, C. crescentus* (Katayama et al., 2010), DNA repair in *E. coli* (Rangarajan et al., 1999), toxin-mediated replication fork collapse in *C. crescentus* (Aakre et al., 2013). All described β-clamp interaction partner proteins share similar motif, the Clamp Binding Motif (CBM; Dalrymple et al., 2001).

### β-clamp loading at *origin* of iteron plasmid

**Figure 4:**
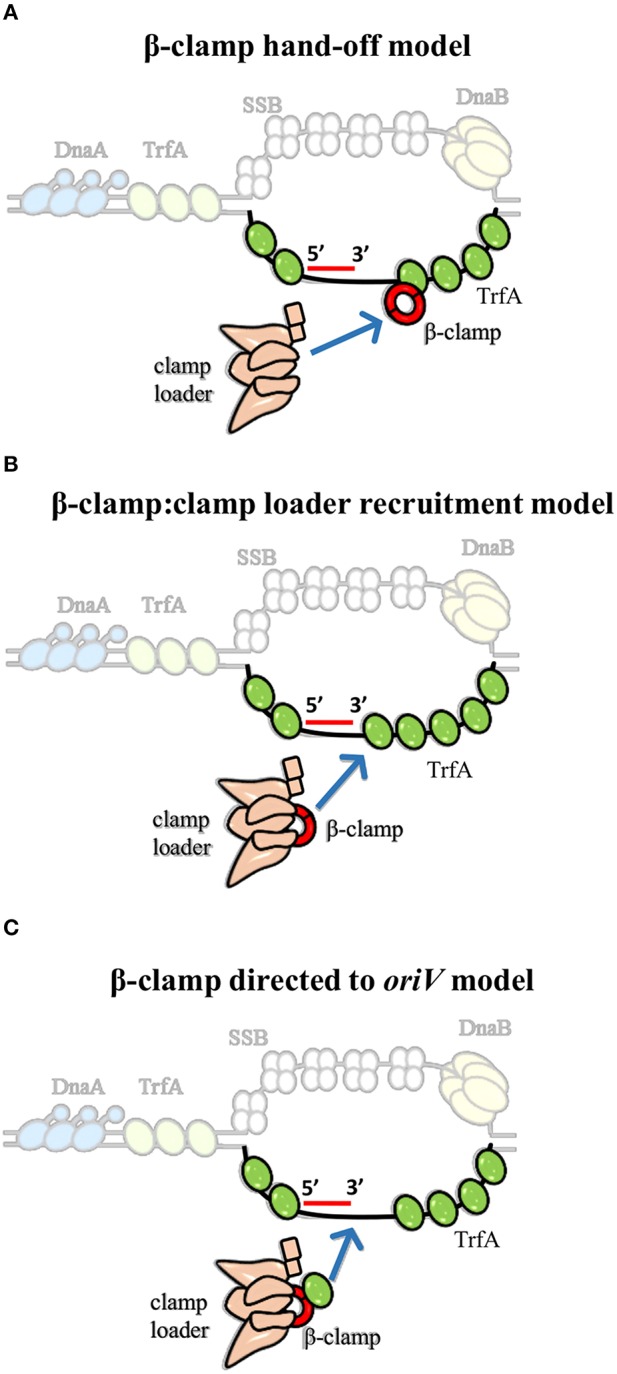
**Models for the contribution of TrfA to β-clamp loading at RK2 plasmid ***origin*** (***oriV***)**. We propose three model mechanisms for β-clamp loading at *oriV* through cooperated action of TrfA and clamp loader. **(A)** The first model suggests that ssDNA-bound TrfA recruits and hands off the β-clamp to clamp loader. **(B)** The second model implies that ssDNA-bound TrfA recruits the β-clamp in complex with clamp loader, thereby increasing the local concentration of β-clamp:clamp loader complex at *oriV*. **(C)** The third model indicates that TrfA binds β-clamp that is in complex with the clamp loader and directs it to the ssDNA of *oriV*.

Interestingly, clamp binding motif was also identified in plasmid replication initiators, including RK2 plasmid initiator-TrfA (Kongsuwan et al., 2006; Dalrymple et al., 2007). It was shown that TrfA protein lacking the leucine 137 and phenyloalanine 138 within the clamp binding motif is unable to bind β-clamp (Kongsuwan et al., 2006). The TrfA ΔLF mutant facilitated the determination of biological relevance of this interaction. The complex of TrfA and β-clamp was found to be the key feature for replisome assembly and thereby for *oriV-*dependent DNA replication of both supercoiled dsDNA plasmid and ssDNA plasmid *in vitro*, albeit the clamp loader complex is still crucial (Wawrzycka et al., 2015). Hence, the question arises—how do the Rep and clamp loader cooperate to load the β-clamp at plasmid *origin*? Three hypothetical models to explain the mechanism of Rep-mediated β-clamp loading could be considered (Figure 4). In the “β-clamp hand-off model” TrfA binds to the bottom strand of ssDNA close to the 3′ end of synthesized primer, recruits β-clamp, and hands it off to the clamp loader complex (Figure 4A). Then, the δ subunit of clamp loader opens the β-clamp and clamp loader positions it onto primer-template junction, as it is thought to occur during replisome assembly at *E. coli oriC*. This model is consistent with the results of the *in vitro* DNA replication experiments performed with the use of ssDNA, containing sequence of RK2 plasmid *oriV* (Wawrzycka et al., 2015). It was demonstrated that TrfA interacts with specific strand of ssDNA of DUE, i.e., the bottom strand, which serves as the site for replisome assembly (Wegrzyn et al., 2014; Wawrzycka et al., 2015). It can be further speculated that TrfA may assist the clamp loader in recognition of the 3′ end of primer-template junction within the *oriV*. Another possible role of TrfA is illustrated in Figure 4B (second model, “β-clamp:clamp loader recruitment model”). Once TrfA is bound to bottom single strand of DUE, it recruits the β-clamp, which is in complex with clamp loader, to the RK2 plasmid *origin*. Thus, the local concentration of β-clamp:clamp loader complex increases, the clamp loader can assemble β-clamp onto the 3′ end of a primer within the plasmid *origin*. Because TrfA-β-clamp interaction was shown in the absence of DNA [using both ELISA and SPR (Surface Plasmon Resonance) technique (Kongsuwan et al., 2006; Wawrzycka et al., 2015)], the third model may also be justified (Figure 4C, “β-clamp directed to *oriV* model”). In the third model the TrfA that is not bound to DNA forms complex with β-clamp associated with the clamp loader, then directs it to the plasmid *origin, oriV*. Next, the clamp loader:β-clamp:TrfA complex binds to the bottom strand of DUE via TrfA. TrfA passes the β-clamp bound to clamp loader on the primer-template junction. Although ATP binding to clamp loader (namely γ and τ subunit) is required for β-clamp opening, it cannot be excluded that TrfA—whose ATPase activity has not been revealed—substitutes the clamp loader's function at this stage. TrfA may capture β-clamp in open conformation and load it onto primed DNA. Since ATP hydrolysis is required for β-clamp closing (Trakselis et al., 2001), here may participate the clamp loader.

## DNA synthesis and the role of SSB

After the β-clamp closes around primer-template junction and clamp loader dissociates, the final replisome component arrives—the Pol III core, that is composed of three subunits: α (DNA polymerase), ε (3′–5′ proofreading exonuclease) and θ (ε subunit stabilizer; Kelman and O'Donnell, 1995; Taft-Benz and Schaaper, 2004). The number of Pol III cores within the replisome strictly depends on the clamp loader composition, since Pol III core is connected only through τ subunit to the clamp loader. Various clamp loader complexes were widely studied in the light of processivity of DNA replication and it was established that three ATPases (τ or γ subunit) must be included with δ and δ' subunits to form active pentameric structure (Kelch, 2016). Initially, it was thought that clamp loader contains two τ subunits (τ_2_γ*δδ*'χψ), so that two Pol III cores could constitute the replisome and synthesize the leading strand and lagging strand at the same time (Maki et al., 1988). However, further reports have argued on the stoichiometry of hPol III subunits (McInerney et al., 2007; Reyes-Lamothe et al., 2010; Dohrmann et al., 2016). The millisecond single molecule fluorescence microscopy as well as *in vitro* biochemical experiments showed that active *E. coli* replisome contains three molecules of polymerase that are functional at replication fork (McInerney et al., 2007; Reyes-Lamothe et al., 2010). Both these studies assumed that trimeric polymerase is associated with three molecules of τ subunit. However, the very recent data indicated that in a bacterial cell there is predominately present Pol III_2_τ_2_γ*δδ*'χψ complex (Dohrmann et al., 2016). Since plasmids do not encode all essential proteins required for a plasmid replication, it is implied that the stage of DNA synthesis is similar during chromosomal DNA replication.

The DNA synthesis is facilitated by SSB, especially on the lagging strand (where the DNA synthesis is performed discontinuously) and is present in organisms from all domains of life (Shereda et al., 2008). Primary function of SSB is to protect ssDNA against degradation and melting secondary structures (Mackay and Linn, 1976; Meyer et al., 1979). SSB is linked to the clamp loader via χ subunit (Glover and McHenry, 1998), which was shown to be important for DnaG primase displacement (Yuzhakov et al., 1999). Yet, SSB was termed the organizer of genome maintenance complexes and was shown to interact with at least 14 proteins, thus implying its diverse functions (reviewed in details in Shereda et al., 2008). The SSB interactions with proteins involves the C-terminal region of SSB, that is highly conserved among eubacterial SSB proteins. Some plasmids also encode SSB-like proteins, i.e., plasmid F, ColIb-P9, and RK2 (Chase et al., 1983; Howland et al., 1989; Thomas and Sherlock, 1990). While SSB of plasmid F and ColIb-P9 have similar structural domains, the RK2 SSB, termed P116, is smaller and contains only the N-terminal domain, which is responsible for DNA binding. P116 lacks the C-terminal protein binding-tail (Curth et al., 1996; Naue et al., 2013; Su et al., 2014), which may suggest that the role of P116 limits to ssDNA protection against nucleases.

## Conclusions and perspectives

The ground-breaking model of DNA replication initiation, introduced by Bramhill and Kornberg is still valid today (Bramhill and Kornberg, 1988b). They proposed that first the DnaA binds to DnaA-boxes to form an initial complex, then DnaA melts the AT-rich region (DUE) to form an open complex. Finally, DnaA directs the DnaB:DnaC complex into the open complex, thus forming a pre-priming complex, which marks the future forks of DNA replication (Bramhill and Kornberg, 1988a,b). In this concept the chromosomal replication initiator, DnaA triggers the DNA replication initiation and is further required at each stage of the replication initiation process. Iteron plasmids also encode replication initiators that drive their replication initiation machinery. Despite the fact that plasmid and chromosomal replicons use overlapping set of proteins, there seems to be some subtle differences that may largely affect the whole process. Recent reports describe novel functions of replication initiators, both plasmid and chromosomal, that outreach the replication initiation process. The contribution of plasmid Rep protein to replisome assembly by providing direct Rep-β-clamp interaction, shed a new light on how far-reaching activities replication initiators have i.e., determination of direction of DNA replication (Wawrzycka et al., 2015). DnaA is also involved in a regulation of DNA replication initiation by a process termed RIDA (Regulatory Inactivation of DnaA; Katayama et al., 2010). One may ask if there is any other unanticipated activity of replication initiators to be discovered? What other processes are influenced by replication initiators? Described model mechanisms and unsolved questions of the structure-function relation of replication initiators in DNA replication and beyond this process await to be experimentally challenged.

## Author contributions

KW and MG wrote the manuscript and prepared figures, UU prepared the model of *E. coli* DnaA and figures, IK discussed and corrected the text of the manuscript.

### Conflict of interest statement

The authors declare that the research was conducted in the absence of any commercial or financial relationships that could be construed as a potential conflict of interest.
